# Factors Associated with Awareness, Attitudes and Practices Regarding Common Eye Diseases in the General Population in a Rural District in Bangladesh: The Bangladesh Population-based Diabetes and Eye Study (BPDES)

**DOI:** 10.1371/journal.pone.0133043

**Published:** 2015-07-22

**Authors:** Fakir M. Amirul Islam, Rahul Chakrabarti, Silvia Z. Islam, Robert P. Finger, Christine Critchley

**Affiliations:** 1 Department of Statistics, Data Science and Epidemiology, Swinburne University of Technology, Hawthorn, Australia; 2 Organisation for Rural Community Development, Dariapur, Narail, Bangladesh; 3 Centre for Eye Research Australia, Royal Victorian Eye and Ear Hospital, University of Melbourne, Melbourne, Australia; 4 School of Economics, Finance and Marketing, RMIT University of Technology, Melbourne, Australia; LV Prasad Eye Institute, INDIA

## Abstract

**Background:**

To assess the awareness, attitudes, and practices associated with common eye diseases and eye care utilization in a rural district of Bangladesh.

**Methods:**

Data were collected using a multilevel cluster random sampling technique from 3104 adults aged ≥30 years from the Banshgram union with a questionnaire assessing the awareness, attitudes and practice about diabetes and common eye diseases, educational attainment, socio-economic status, and medical history.

**Results:**

Participants were aged between 30 and 89 years with a mean (SD) age of 51 (12) years and 65% were female. The majority of participants had heard of cataracts (90%), trachoma (86%) and Pterygium (84%), yet only 4% had heard of diabetic retinopathy (DR), 7% of glaucoma and 8% of Age-related macular degeneration (AMD). However, 58% of participants did not know vision loss could be prevented. Factors associated with lower awareness regarding common eye diseases were increasing age, lack of formal schooling, and lower socio-economic status. A lower proportion (57%) of people with no schooling compared to those who had attained at least secondary school certificate education (72%) reported that they knew that vision loss could be prevented (p<0.001). Overall 51% of people had heard of at least six (67%) out of nine items relating to awareness of common eye diseases. This included 41% of participants aged 65 years or older compared to 61% of those aged 30–35 years (p<0.001). Only 4% had an eye check at least once a year and higher education and better SES were associated with higher frequency of eye checks.

**Conclusions:**

In rural Bangladesh awareness of cataract, trachoma and pterygium was good but limited in relation to the potentially blinding conditions of glaucoma, DR, and AMD. The results show a large gap between public awareness and treatment practices about common eye diseases. Public health promotion should be designed to address these knowledge gaps.

## Introduction

Globally, 191 million people were estimated to have moderate and severe vision impairment (MSVI), with 32.4 million including 60% women being blind. Although the age-standardized prevalence of blindness in older adults of those aged ≥50 years in South Asia was lower than that of the highest in Sub-Saharan Africa (4.4% vs. 6.0%), the prevalence of MSVI was the highest in South Asia (23.6%) compared to the second highest in Oceania (18.9%). [1] The major causes of visual impairment are uncorrected refractive errors (43%) followed by cataracts (33%). Other causes of visual impairment include glaucoma, diabetic retinopathy (DR) and age-related macular degeneration (AMD). [2] It is plausible that the epidemiology of reversible vision loss in Bangladesh is related to an increasing trend in non-communicable diseases particularly diabetes, and other trends in lifestyle-related factors including modification of diet, sedentary lifestyle, and smoking. [3] A nation-wide population based study of adults ≥ 30 years of age in Bangladesh reported 22.6% with low vision [4] and 22.1% with myopia. [5] A growing body of evidence from Knowledge, Attitudes and Practice (KAP) studies have supported the need for greater awareness of prevention, diagnosis, risk factor control and disease management. [6, 7] It is imperative to reduce the burden of these diseases through the implementation of public health policy by identifying the risk factors of visual impairments. A major factor hindering public health strategies is a lack of awareness of eye conditions [8–12] which has been shown to be associated with poorer outcomes in terms of prevention, [9, 10] eye care use, [10, 13, 14] and treatment. [9, 10, 14]

There are a few studies conducted in Bangladesh that report the prevalence and risk factors of visual impairment, but all were conducted more than a decade ago. [4, 5] However, no study has reported awareness, attitudes and practice regarding common eye diseases, their risk factors or management in a general population either in a rural or in an urban city. One exception was in 2011, where Muhit et al.[15] conducted a KAP survey of parents in rural districts of Bangladesh in relation to blindness amongst children. They reported that approximately 75% of the parents knew that vitamin-A deficiency was the leading cause of blindness, more than one quarter believed that eye infection was an important cause of childhood blindness and half of the parents believed that childhood cataracts were untreatable. [15] This has important implications for planning healthcare services.

In this study, we report awareness, attitudes, and practices regarding common eye diseases, their risk factors and the variation according to socio-demographic factors in a rural district in Bangladesh. The study will inform the need for increasing health literacy regarding common eye diseases in the rural areas in Bangladesh.

## Materials and Methods

### Study Sample

The sample consists of 3104 participants aged ≥30 years from the general population of the Banshgram Union in Narail district. They were recruited using a multilevel cluster random sampling from each of the total 18 villages in this rural district from December 2012 to March 2013. The sample size was based on the prevalence of diabetes in adults in Bangladesh of 6.3% in 2012 estimated by the International Diabetes Federation’s Diabetes Atlas. [16] The sample was sufficiently large enough to detect a 3% difference in the proportion of attaining awareness or attitudes related to common eye diseases between males and females, and those with no schooling and primary or secondary level of education (statistical power > 80%, p = 0.05). The study location is approximately 200 km southwest of the capital city Dhaka and has an adult population aged 30 years or above of approximately 5,500.[17] The population density of the study location was 722 per km^2^ compared to the population density 873 per km^2^ in rural districts in Bangladesh. The study location was selected as it was considered to be typical of a rural demographic in Bangladesh. Narail has a population literacy rate of 48.6%, which is comparable to the national literacy rate of 51.8%.

### Recruitment Strategy

The recruitment strategy involved identifying participants aged ≥ 30 years from each of the selected households within 18 clusters of villages. This age range was selected as previous studies have demonstrated that this is an acceptable threshold of age above which the prevalence of diabetes increases. [18] Recruitment started from the far east corner of a village and selected every second households using a systematic random sampling technique and continued until at least 50% of the total eligible adults were interviewed from each of the villages. All eligible adults from the same family were recruited and they were required to attend data collection over two days. On day one, the participants were interviewed using a semi-structured questionnaire following a door-to-door recruitment strategy which took approximately 35 minutes to interview each participant. The interview assessed participants’ awareness, attitudes and practice about diabetes and common eye diseases and other socio-demographic factors including level of educational attainment, and socio-economic status (SES). After interviewing the participants, the data collectors informed participants to attend the nearest community center or school on the next morning (day two) for clinical examination including measuring height, weight, blood pressure and the measurement of fasting capillary glucose. There were four teams of trained data collectors with 4–5 members in each team who received training from the same medical doctor interviewed the participants on day one and collected anthropometric and other measurements on day two. Different teams worked independently. All team members participated in an intensive two day training program before the commencement of the survey. Excluded were those younger than 30 years, and those who were acutely unwell to attend the centre for clinical examination on day 2. Less than 15% of participants interviewed on day one failed to present for day two assessment, with a participation rate above 85%. For this particular study, we have used data collected only from day one based on the household survey. Therefore, it is expected that there should not be any effect on our results from participation or non participation on day 2.

### Questionnaire

A detailed interviewer-administered questionnaire was developed to collect socio-demographic data and to assess participant awareness, attitudes and practice regarding diabetes and common eye diseases. For eye disease, awareness was operationalized by whether or not participants had heard about the disease (nine items), attitudes by their evaluation of treatment if they had a disease (one item), and practice was ascertained based on how frequently they were checking their eyes (one item). The items assessing awareness, attitudes and practice of eye diseases were adapted from a validated instrument used in Cambodia to assess KAP of common eye diseases. [19] The questionnaire was translated into Bengali separately by a local senior educator as well as by the principal investigator. The two versions were then combined and finalised with an agreement on the translated version, using local language for a better understanding by participants where needed. For example, “trachoma” is known as “chok utha” in the local language which is familiar to most of the people. Therefore, “chok utha” was used instead of “trachoma” in the Bengali questionnaire. Five women and five men who are not included in the study were asked to assess comprehension, wording, and appropriateness of the questionnaire before it was finalised. The questionnaire is presented in detail elsewhere. [20] Questions assessing awareness of and attitudes to common eye diseases or symptoms had binary responses options (Yes/No) and questions assessing practice related to eye care had the following response options: Frequently (more than once a year), Regularly (at least once a year), Whenever I have an eye problem, and Never.

The socio-demographic data included gender, age in categories (Less than 35, 35–44, 45–54, 55–64, above or equal 65 years), educational level in categories (no schooling, primary school 1–5 years, high school 6–10 years, Secondary School Certificate (SSC) or above) and socio-economic status (SES). Since most participants had no taxable income a crude measure of SES was used following Cheng et al.[21] asking whether "over the last twelve months, in terms of household food consumption, how would you classify your socio-economic status?" The possible answers were: (i) Insufficient funds for the whole year, (ii) Insufficient funds some of the time, (iii) Neither deficit nor surplus (balance), and (iv) Sufficient funds most of the time. We have reported the study sample, recruitment strategy, data collection, awareness, attitudes and practice questionnaire and socio-demographic factors in detail elsewhere. [20, 22, 23]

### Statistical analysis

Participant’s age, level of education and SES were compared across gender using Chi-square tests. Chi-square tests were also used to detect any differences in awareness across gender, age categories, level of education and SES. The Chi-square Linear-by-Linear Associations option was used to present p values for ordinal categorical trends such as level of education. The participants were categorised based on the number of items out of nine they had heard about, such as at least 5 items, at least six items and so on and the association of socio demographic factors with the categorical outcome of at least six items vs. less than six items was reported using Chi-square tests.

Logistic regression techniques were used to report odds ratios (OR), and 95% confidence interval (CI) for positive attitudes towards treatment of eye diseases adjusting for age, gender, religion, level of education and SES. Socio-demographic factors were also compared for practice of regular eye check using chi-square tests. Statistical software SPSS version 21(IBM SPSS, Armonk, NY, USA) was used for all analyses.

## Ethics approval

We conducted the research in accordance with the tenets of the Declaration of Helsinki and approval was taken from the Human Research Ethics Committee for the Bangladesh Medical Research Council (Reference: BMRC/NREC/2010-2013/68). All participants provided written or verbal consent prior to inclusion in the study. The data collectors signed the consent form for the participants with their approval in the case of when verbal consent was taken. The ethics committee approved this consent procedure. Participants were informed of their privileges to withdraw from the study at any stage if they wanted or to restrict their data from the analysis.

## Results

Of the 3104 participants aged between 30–89 years (mean 51 years and standard deviation 12 years), 65% were females, 84% Muslim and the rest 16% were Hindu. Just under half (47%) had no formal education, 14% had insufficient funds all the time and only 7% had surplus yearly income. The proportion of female at the national level is 50% compared to our recruitment of 65%. Eight percent of participants from our study sample were below 35 years of age compared to 17% at the national level but the other characteristics, such as level of education, religious affiliation and SES of the study participants were similar to those at the national level (Table 1). Since the majority of the non-respondents were males who failed to attend clinics for blood glucose test on day two, no impact on our results is expected because we are not reporting on diabetes or hypertension. Moreover, no significant difference in awareness, attitudes or practice is observed between males and females. Therefore, non-participation is unlikely to have any impact on our conclusions.

**Table 1:** Socio-Demographic Characteristics of the Study participants, and participants from a national representative sample.

	Bangladesh, N = 15.78 (million)	Study sample N = 3104	
	%	N	%
Gender[27]			
Female	49.9	2032	65
Male	50.1	1072	35
*Age, years[27]			
Below 35	17	240	8
35–44	31	876	28
45–54	23	942	30
55–64	15	590	19
Above or equal 65	14	456	15
Religion [27]			
Muslim	88	2599	84
Hindu	11	505	16
†Level of Education [28]			
No Schooling	48	1462	47
1–5 primary	27	921	30
6–10 High school	17	495	16
SSC or above	7	226	7
†Socio-economic status[28]			
Insufficient funds most of the time	19	424	14
Insufficient funds some of the time	39	1077	35
Balance	21	1320	43
Sufficient funds most of the time	21	268	9

*The percentage of people in different age groups at national level has been calculated based on the total people of aged above 30 years

† data are from a national representative sample of size 7541 of aged 35 years and older. The definition of SES is different for two different studies. Insufficient funds most of the time (14%) can be comparable with the poorest (19%); insufficient funds some of the time (35%) can be compared with the poorer to middle class; balance plus sufficient funds most of the time (51%) can be compared with the richer and richest (42%).

### Awareness Assessment

The awareness of eye diseases varied between 4% to 91% depending on the eye condition. In this sample, 91% of participants had heard of cataracts; yet only 4% had heard of DR, 7% heard of glaucoma and 8% heard of AMD. More than 85% of all participants had heard of common eye diseases, however, only 4–11% had heard of common blinding posterior segment diseases. There was no significant difference between gender in awareness in any of the eye diseases except for a slight difference in AMD (11% females vs. 13% males, p = 0.05). Overall older people, and those with no schooling and lower SES were significantly less aware of all the diseases compared to younger, more educated participants and those from higher SES backgrounds. For example, 80% of people aged 65 years or above compared to 94% of people under 35 years (p<0.001); 84% with no schooling vs. 92% with SSC or above educational level (p<0.001) were aware of trachoma. One hundred and two (3.3%) people had not hear of any of the items, 51% of people had heard of at least six 6 (67%) items regarding awareness of eye diseases, and 0.5% had heard of all items (Tables 2 and 3).

**Table 2:** General awareness (known or heard about) of common Eye Disease by Gender, Religion and Age groups, (N = 3104).

			Gender					Religion					Age groups*				
	Total		Female (N = 2032)		Male (N = 1072)			Muslim (N = 2599)		Hindu (N = 505)			Age, <35 (n = 240)		Age, > = 65 (N = 456)		
	N	%	n	%	N	%	P	N	%	N	%	P	N	%	n	%	P for trend
Red eye	2698	87	1772	87	926	86	0.47	2328	90	370	73	<0.001	221	92	374	82	<0.001
Blurred vision	2795	90	1836	90	959	90	0.44	2405	93	390	77	<0.001	224	93	396	87	<0.001
Cataract	2809	91	1830	90	979	91	0.27	2400	92	409	81	<0.001	226	94	401	88	0.002
Trachoma	2655	86	1740	86	915	85	0.71	2271	87	384	76	<0.001	226	94	365	80	<0.001
Glaucoma	219	7	144	7	75	7	0.94	189	7	30	6	0.43	10	4	20	4	0.30
Diabetic Retinopathy	124	4	76	4	48	4	0.32	105	4	19	4	0.82	7	3	14	3	0.30
Age Related Macular Degeneration	360	12	219	11	141	13	0.05	296	11	64	13	0.05	26	11	49	11	0.27
Pterygium	2593	84	1689	83	904	84	0.42	2168	84	425	84	0.93	212	88	367	81	0.008
Vision loss can be prevented	1812	59	1158	57	654	61	0.03	1605	62	207	41	<0.001	164	68	243	54	<0.001
At least six items known	1567	51	1000	49	567	53	0.05	1389	53	178	35	<0.001	145	61	185	41	<0.001

**Table 3:** General awareness (known or heard about) of common Eye Disease by level of Education and Socio-economic status (N = 3104).

	Education level									Socio-economic status*								
	No Schooling (N = 1462)		Primary (N = 921)		Secondary (N = 495)		SSC or above (N = 226)		P for trend	Very poor (N = 423)		Poor (N = 1077)		Middle class (N = 1320)		Rich (N = 268)		P for trend
	n	%	n	%	n	%	n	%		n	%	N	%	N	%	n	%	
Red eye	1236	85	809	88	443	89	210	93	<0.001	329	78	909	84	1209	92	240	90	<0.001
Blurred vision	1290	89	842	91	447	90	216	96	0.002	355	85	951	88	1231	93	245	91	<0.001
Cataract	1283	88	844	92	466	94	216	96	<0.001	352	84	948	88	1249	95	245	91	<0.001
Trachoma	1221	84	792	86	435	88	207	92	<0.001	329	78	889	83	1191	90	231	86	<0.001
Glaucoma	79	5	88	10	39	8	13	6	0.10	33	8	80	7	80	6	25	9	0.73
Diabetic Retinopathy	46	3	43	5	20	4	15	7	0.02	23	5	37	3	44	3	18	7	0.98
Age Related Macular Degeneration	123	8	150	16	52	11	35	15	0.001	45	11	162	15	108	8	42	16	0.20
Pterygium	1187	81	789	86	414	84	203	90	0.002	308	73	875	81	1164	88	233	87	<0.001
Vision loss can be prevented	826	57	512	56	312	64	162	72	<0.001	206	49	604	56	828	63	166	62	<0.001
At least six items (67%) known	677	46	473	51	269	54	168	66	<0.001	144	34	509	47	754	57	155	58	<0.001

*Insufficient funds most or all of the time = very poor, Insufficient funds some of the time = poor, Balance = middle class, and Sufficient funds most or all of the time = Rich.

### Attitudes

The attitudes towards taking treatment for eye disease were very positive. A total of 2777 (90%) people were in favour of taking treatment for eye disease, and there was no difference in attitudes between gender, age groups, or level of education. However, the people with sufficient funds most of the times were almost twice more likely to have positive attitudes towards treatment compared to those with insufficient funds most or all of the time, odds ratio (OR) 1.90, 95% confidence interval (CI) (1.09, 3.30) (Table 4). There was no significant interaction between awareness and attitudes.

**Table 4:** Associations of socio-demographic characteristics with attitudes (Should they seek treatment for eye diseases).

	No		Yes		
	n	%	n	%	OR (95% CI)*
Total	320	10.3	2777	89.7	
Less than 35 years	30	12.6	208	87.4	1.0
35–44	71	8.1	803	91.9	1.53 (0.96, 2.44)
45–54	92	9.8	849	90.2	1.24 (0.78, 1.96)
55–64	70	11.9	520	88.1	1.01 (0.62, 1.63)
Above or equal to 65 years	57	12.6	397	87.4	0.97 (0.59, 1.62)
Gender					
Female	218	10.8	1808	89.2	1.0
Male	102	9.5	969	90.5	1.20 (0.92, 1.56)
Religion					
Muslim	248	9.6	2346	90.4	1.0
Hindu	72	14.3	431	85.7	0.61 (0.46, 0.82)
Education level					
No Schooling	154	10.6	1304	89.4	1.0
Primary (1–5)	98	10.7	821	89.3	0.93 (0.71, 1.23)
Secondary (6–10)	53	10.7	441	89.3	0.89 (0.62, 1.27)
SSC or above	15	6.6	211	93.4	1.37 (0.77, 2.46)
SES					
Insufficient funds most or all of the time	59	14.1	360	85.9	1.0
Insufficient funds some of the time	86	8.0	989	92.0	1.87 (1.31, 2.66)
Balance	149	11.3	1171	88.7	1.25 (0.90, 1.74)
Sufficient funds most of the time	20	7.5	247	92.5	1.90 (1.09, 3.30)

*Odds ratio (95% confidence interval) adjusted for age (except for age), gender (except for gender), religion (except for religion), level of education (except for level of education) and SES (except for SES).

### Practice (eye examination)

Ninety-six percent of participants in the study area had never had a previous eye examination. Two percent of people reported having had an eye check within the previous year, and another 2% reported having 2–3 eye checks per year. Of those who had regular eye checks, 62% reported to have an eye problem. People with secondary school certificate (SSC) or above level of education (4.9%) compared to no schooling (1.5%), (p<0.001), and people with sufficient funds most of the time (6.0%) compared to people with insufficient funds (1.4%) (p = 0.002) had significantly higher frequency of at least two eye checks per year (Table 5). Based on the self-reported questionnaire used in the study, twenty eight percent of participants reported having a history of eye disease or a problem with their vision. However, only 5% of them had an eye check at least once in a year. This compared to 3% who did not have any self-reported history of eye disease. With regards to patients with diabetes (n = 222), 9% had at least one eye check per year, compared to 3.0% of participants without diabetes.

**Table 5:** Associations of socio-demographic characteristics with eye check per year.

	No at risk	Once in a year		At least twice in a year		P for trend
	N	n	%	n	%	
Total	3104	49	1.6	70	2.3	
Age, years						0.08
Less than 35 years	238	4	1.7	2	0.8	
35–44	878	9	1.0	18	2.1	
45–54	942	17	1.8	21	2.2	
55–64	590	11	1.9	18	3.1	
Above or equal to 65 years	456	8	1.8	11	2.4	
Gender						0.84
Female	2032	34	1.7	46	2.3	
Male	1072	15	1.4	24	2.2	
Religion						0.91
Muslim	2599	42	1.6	58	2.2	
Hindu	505	7	1.4	12	2.4	
Education level						<0.001
No Schooling	1462	16	1.1	22	1.5	
Primary (1–5)	921	19	2.1	16	1.7	
Secondary (6–10)	495	8	1.6	21	4.2	
SSC or above	226	6	2.7	11	4.9	
SES						0.002
Insufficient funds most or all of the time	424	7	1.7	7	1.7	
Insufficient funds some of the time	1077	19	1.8	15	1.4	
Balance	1320	16	1.2	30	2.3	
Sufficient funds most of the time	268	6	2.2	16	6.0	

## Discussion

To the best of our knowledge, our study is the first population-based study of awareness, attitudes and practice associated with eye diseases amongst a rural population in Bangladesh.

The main findings of this study were that the overall awareness of common eye diseases in a rural community in Bangladesh was poor. Approximately 50% of participants did not know that vision loss could be treated. This deficiency was more pronounced amongst people with no schooling, lower SES and older people. The usefulness of the results is two-fold. Firstly, we have demonstrated a significant deficiency in basic awareness of common eye diseases in a rural area regardless of socioeconomic status. Secondly, we have shown that people with better socio-economic status and higher basic educational attainment backgrounds are more inclined to have eye checks. This strongly suggests that increasing health literacy regarding eye disease is required at all socioeconomic levels to improve attendance and health care seeking practices. This is particularly relevant in developing countries such as Bangladesh where there is a high concentration of ophthalmic services at the tertiary level. Whilst it was beyond the scope of this study to demonstrate the relevance of our findings to the prevalence of eye disease, we believe that increasing understanding and acceptance of the importance of regular eye examination may reduce visual impairment and overall cost of eye care. [11]

This coincides with our previous research from the same sample that showed a lack of awareness about diabetes, its risk factors and management. [22] Furthermore similar trends were found here in relation to educational level and socio-economic status, where higher awareness of diabetes was found among people who were more educated or had a better socio-economic status. This trend is consistent with similar associations reported for health literacy in both, the developing, [11, 12, 22, 24] and the developed world. [10, 25, 26] Interestingly, gender inequity in awareness of eye diseases was not found to be a significant problem though a significantly higher level of awareness about diabetes and its risk factors was found in males from the same sample. [22]

Though we have collected data from a single location, the location can be considered to be a typical rural area of Bangladesh. We have compared the characteristics of our study participants with national level data [27] and a nationally representative sample [28] and found them to be very similar. Thus, our data can be considered as a true presentation of the typical rural areas of Bangladesh and establishes a baseline for understanding perceptions of eye health within rural Bangladesh. The level of awareness of common eye disease was also found to be similar with results from neighbouring India and Nepal [11, 12].

The study demonstrated that attitude towards treatments of eye diseases amongst the rural cohort is very positive. Despite this, it appears that access to eye examination remains a challenge amongst the population. Evidence suggests that increasing public awareness has significantly reduced the lag to diagnosis among patients with rheumatoid arthritis in United Arab Emirates.[29] and successfully increased participation in breast cancer screening in Hawai’i.[30]

The majority of participants who reported an eye problem or disease did not have an eye check which raises a pertinent concern regarding the influence of health promotion interventions and resource allocation on change of practice. This is of particular concern given the awareness amongst the cohort overall was poorest particularly for insidious conditions such as glaucoma, DR, and AMD in which symptoms of vision impairment often present late in the disease course. Studies from diabetic retinopathy in low resource settings have shown that patients tend to present only when disease becomes sight-threatening or there is a sudden deterioration in vision. [31] Incidentally our study showed there was an almost three-fold increase in at least one eye examination per year amongst people with diabetes compared to those without. Whilst this can be expected, it highlights the imperative upon primary health care practitioners in emphasizing regular eye checks as part of a general health assessment. Thus, health promotion must not only emphasise eye examination when symptoms develop, but more importantly upon the necessity of regular eye checks to prevent development of sight-threatening changes. However, from the perspective of patient prognosis and health-economic ramifications sight-threatening disease can be prevented with timely referral for eye examination. [32] The opportunity to test presenting visual acuity and appropriately refer for formal vision assessment may first present itself at the primary care level as part of a general health evaluation.

Our study is the first reliable data on the awareness, attitudes and practice of common eye disease and their risk factors in the general population in Bangladesh, especially among rural people. Strengths are data collection through face-to-face interviews from a large number of adults. The potential drawback of our study is a report from a single-occasion collection of data from a single location. Whilst we have attempted to capture the situation in Banshgram, the study would obviously need to be repeated in a random sample of other remote areas in order for the results to be truly representative of a national perspective. Data were also collected by a number of teams but we were unable to check the inter or intra interviewer’s reliability. The resources did not allow for two interviewers to assess the same individual meaning inter-rater reliability could not be assessed. However, the interviewers were trained to interview participants using the same approach. A pilot study was also conducted to check the questionnaire to assess it comprehension, wording, and appropriateness. Further adjustment to the questionnaire was needed. We collected data from all eligible adults from the same family expecting a cluster effect, but we had not recorded how many times more than one participant was from the same family. However, we would require a sample size of 1356 participants to observe a significant p value with a statistical power of at least 80% for a minimum of 3% difference in attaining awareness of particular disease items between gender or level of education. If we adjust for cluster effect by 1.5 times, our required sample size would be 2048. However, we have our current sample size of 3104 participants.

## Conclusions

The study demonstrated that there is overall poor understanding of sight-threatening eye conditions amongst a rural cohort in Bangladesh. Of most concern was the poor understanding of avoidable sight-threatening eye diseases, particularly DR and glaucoma. Our findings suggest that health literacy and public health interventions for eye care should target certain groups of the population particularly women and older people, those with lower education and socioeconomic status.

## Supporting Information

S1 Data BPDES data KAP eye. (XLS)
